# Case of Dracunculus, or Filaria Medinensis

**Published:** 1830-10

**Authors:** Gilbert T. Burnett

**Affiliations:** Surgeon, &c.


### DRACUNCULUS.

*Case of Dracunculus, or Filaria Medinensis.* Communicated by GILBERT T. BURNETT, Esq. Surgeon, &c.

As Guinea worms, although familiar to surgeons who visit the coasts of Africa, and to practitioners in our settlements in either India, are not frequently met with in this country, perhaps the following case, the progress of which was investigated and watched with considerable attention, both before and during the time the patient was in St. George's Hospital, may not be wholly devoid of interest; and, if so, it may be probably esteemed not unworthy a place among the select records of the London Medical and Physical Journal.

Anne Tennerson, aged fifty, a soldier's wife, and the mother of five children, returned to England on the 1st of May, after a residence in India of upwards of eighteen years. During her sojourn abroad, she had in general enjoyed very good health; the only exceptions being an attack of the ordinary fever of the country, and another of cholera morbus, upwards of ten years since, from both which she had entirely recovered. She embarked at Bombay in December, i. e. nine months ago, in perfect health. In the course of about two months after the commencement of the voyage, and when off the Cape, she was seized with very severe pains of the left leg and foot, which she appeared totally unable to describe; for of them only a negative character can be obtained, viz. that "they were not at all like rheumatism," to which it would seem they had been at first attributed. She had never been affected with Guinea worm while in India, and not the least suspicion was entertained of Dracunculus being the cause of her sufferings. There was no apparent swelling of the leg, nor any external visible disorder. The pains, however, continued, and, in spite of the means employed by the surgeon on board, and others after her arrival in England, for their relief, they gradually increased, and at times were extremely acute: indeed, during the last three months, they have been almost constantly present.

Early in June, i. e. about a month after her arrival, she first noticed several small lumps, or slight elevations of the integuments, which became inflamed, and looked as if they would fester; but it was not until August that she observed a thready elongation stretching from one of these tubercles, which line might be traced in its extension under the skin for two or three inches. From this she immediately conjectured it to be a Guinea worm, having seen such symptoms in other persons abroad; but previously she had no idea what ailed her.

Having walked much on the 10th of September, the following morning, when about drawing on her stocking, she found that the swelling on the left foot had burst during the night without pain, or her being aware of it, and that a dracunculus had protruded itself about an inch. She tied its end with a piece of cotton, and, winding it round a card, extracted as much as she says "would reach from her elbow to her fingers' ends." The appearance of the worm is something like a bit of wet catgut; it was wound round some soft lint, and fastened to the skin by strips of adhesive plaster; but, notwithstanding this precaution, on getting into bed she accidentally broke it off close to the surface. There is another pimple on the inner side of the left thigh, just above the knee, and round about it the convolutions of another worm can be distinctly seen and felt underneath the skin. There are two other similar pimples, and several slight indistinct elevations, which are suspected to be the nests of other worms.

September 14th. Inflammation followed the rupture of the worm: this has been relieved by antiphlogistic means, and the pain of the part alleviated by using the foot bath.

16th. Inflammation returned; much pain and redness of the foot and instep, where the worm was broken off; abscess forming. No apparent change in the pimple just above the knee; but, at ten o'clock last night, another worm protruded itself from a small opening formed spontaneously in the upper part of the left thigh, near the pudendum. This pimple had entirely escaped notice, and had not been attended with any marked uneasiness or pain. The appearance of the worm is like that of its precursor. Eight or ten inches have been wrapped round a pledget of lint, and confined by plaster as before.

17th. Abscess in the instep opened.

18th. The worm has again appeared at the incision, and a portion of it has been extracted.

19th. The dracunculus on the thigh broken off; much irritation. Fomentations and poultices applied.

22d. The worm at the instep again given way.

24th. Abscess forming above the ankle.

October 4th. Poultices, &c. have relieved pain, the pus has been evacuated, and the abscesses now are nearly well. In the upper part of the calf of the right leg there is a small, hardish tumor, indicating the presence of another worm; the integuments are inflamed and very tender.

12th. The tumor on the calf of the right leg has much increased in size, and is daily expected to give exit to the dracunculus which is contained within it, and which can be traced by its convolutions beneath the skin. The small tubercle on the left thigh has not at all increased or altered its appearance since the first note of it on the 11th of September.

18th. The swelling on the right calf continued inflamed and very sensible when touched, although the irritation was so much moderated that it was not painful when kept undisturbed, until this morning, when the patient, while fomenting it, found the skin suddenly give way, and a small portion of the contained worm became consequently extruded. Its mode of exit was somewhat different to that of either of the previous ones, the head not appearing first, but the integuments having been ruptured over a convolution, a loop was thus projected, but the head of the animal was easily drawn out, and several inches have been wrapped round lint as heretofore: the worm was said to have resisted the extraction, and that not only a resistance, but a reaction was sensible, while holding it between the fingers, as if it was endeavouring to withdraw itself beneath the cuticle.

20th. The worm has been more and more extracted each morning: it has a beautiful semitransparent hue, something like the lighter shades of the opal.

24th. The parts in the neighbourhood of the worm have been inflaming more and more each day; now they are exquisitely tender, and there is much heat, redness, and swelling. The aperture through which the worm hangs has enlarged, and suppuration is commencing. The worm has lost much of its brilliancy, and it is no longer semitransparent, but resembles wet catgut. Much care has been taken not to break this dracunculus, and it is still entire; but the pain seems to be as great as in the previous cases when the worm was broken. The woman, who does not appear to be at all of a querulous disposition, is often found in tears. Yesterday the tubercle on the left thigh, although very slightly enlarged, gave way, and furnished another worm. This dracunculus is much smaller than either of the foregoing, and has not the brilliant opalescence of the last. Poultices and fomentations, seeming to afford the most relief, are assiduously applied to both.

29th. The worm from the calf of the right leg has not been forcibly broken, but appears to have been killed by the heat of the poultices and fomentations, and to have become disorganized. Portions, like soddened shreds of parchment, are extruded, and brought away with every poultice. The leg is much easier. The other smaller worm on the left thigh is as yet unbroken. The patient walks about a little, but her health has suffered much from the continual irritation which the succession of worms, and their consequent abscesses, have kept up.

November 2d. Symptomatic fever lessened; the worms continue daily to be extracted. She states that, from some peculiar sensations in the left foot, where the dracunculus first appeared, and which has for some time been apparently quite well, she thinks that either some part of the original worm has been left, or that another is lurking therein. When asked to describe these sensations, she says they are unlike any thing she ever felt; also that she thinks she can perceive an indistinct motion, as if the animal attempted to vary its situation.

13th. The worms have both been extracted gradually day by day: the small one progresses very slowly, sometimes not more than half an inch being afforded in twenty-four hours.

19th. The fomentations having been used warmer, the exit of the worm has been rather more free. The right foot is still suspected by the woman, but there is no appearance of a worm.

December 1st. The worms still continue to be extracted day by day, and are now supposed to be entirely removed. The ulcer in the calf of the right leg is healing. The dracunculus from the left thigh measures upwards of three feet in length; but, on examining the end, it has not the gradual acumination nor the caudal hook which characterizes the species. It seems as if the integument had been ruptured, and about an inch of the inner substance of the worm drawn out: probably this was occasioned by the hooked tail retaining it firmly within the wound.

5th. The ulcerations now all healed; health much improved, and the uneasy feelings in the left foot much abated.

No further notes were taken. The patient soon after left town, and continued well for at least a considerable time: most probably the whole of the dracunculi had been removed.

This case would seem chiefly to have differed from the few of Malis Filariæ on record, by the marked severity of the previous symptoms; and, although so many openings were effected by the worm, or more probably by the several worms, a much less extent was drawn out than seems to have been usually afforded by the dracunculi, when occurring in warmer latitudes. In an account published by Messrs. HUTCHESON and FORBES, in the second part of the fifth volume of the Edinburgh Medical Essays, page 269, I find, on reference, they state that, during a voyage from Jamaica to Bermuda, a case occurred to them in the person of a youth from Guinea, fifteen years old, and that they drew out from him a piece of dracunculus ten feet and a half in length; and not only this, but also that in the course of eight weeks, they extracted in several fragments, and from different ulcers, thirty yards of the worm, i. e. the almost incredible quantity of ninety feet!

The Filaria medinensis, or Guinea worm, has long been noticed: the Malis Filariæ is, indeed, a disease of classic celebrity. PLUTARCH quotes Agatharcides for an account of these animals, and calls them δρακοντια, whence the ordinary name *dracunculus*. It would seem that it was once doubted whether they were truly distinct animal existences, or whether they were not rather to be considered as diseased formations of those parts in which they are found. The negative of this latter opinion has been, however, long established, and it probably arose from a misinterpretation of the Arabian physicians; for Dr. Mason Good has observed that the *irk Medini* should have been translated Vermis Medinensis, and not, as it has been corruptly rendered, Nervus or Vena Medinensis. The dracunculi are said to be "frequent in the morning dew," and are supposed then to perforate the cuticle, and bury themselves beneath it, especially in the naked feet of the Negroes. It seems to me, however, from observing the progress of the worms in the foregoing case, to be not improbable that the ova may be deposited beneath the cuticle by the parent, or conveyed into the system with the ingesta; as the insinuation of the mature dracunculus could scarcely escape observation: nor is it likely that it would remain so long in a dormant state as we frequently find that it has done, viz. for months, nay, sometimes for upwards of a year; indeed, authors do state that it has been known to exist in the human body many years without producing pain or inconvenience. Still it must be confessed that, of this opinion I have been unable to procure any decided confirmation; for, on endeavouring to trace the earlier history, I could only learn that, of the eighteen years of this woman's life in India, the eleven first were passed in Guzzurat, and the latter seven at Matungha, about five miles from Bombay; that the water which is used and drank at Matungha is collected and kept in tanks, while that with which they were supplied in Guzzurat and elsewhere is procured from wells and rivers; that in Guzzurat the Guinea worm is unknown, except as brought from the neighbourhood of Bombay; while at Matungha and its vicinity it is very common, appearing on all parts of the body. Indeed, its very frequent occurrence among the soldiers at Matungha has excited the attention of the authorities there, and committees were appointed, which sat for many months to investigate the cause, and to suggest some mode of prevention; but I could not learn that any thing satisfactory had been ascertained, or any important measures decided on.

This woman dwelt somewhat on the circumstance that at Matungha, in addition to the musquittos, which abound in all parts of India, there is a blueish fly, which is described as being peculiar to that neighbourhood, and which, by its biting or stinging, annoys the people very much; but it is not probable that the Filaria should be the larva of any such fly, as it is, or at least appears to be, one of the "Entozoa Nematoidea" of Rudolphi, the "Intestinaux cavitaires" of Cuvier. The genus Filaria, of which several species are known, is distinguished by having a very lengthened and much extenuated body, the anterior extremity of which is terminated by a round mouth; the abdominal cavity contains a long intestinal canal leading to an anus, and the organs of generation are described as being distinct. The most celebrated, and to us the most important, species is the Filaria medinensis; the specific character of which is its hooked and pointed tail.

